# Alopecia management from a comparative perspective: pharmacological and pharmaceutical insights in humans, dogs, and cats

**DOI:** 10.3389/fvets.2026.1907027

**Published:** 2026-08-25

**Authors:** Jieun Seo, Jang-Hyuk Yun

**Affiliations:** 1 Faculty of Engineering, Yokohama National University, Yokohama, Kanagawa, Japan; 2 Kanagawa Institute of Industrial Science and Technology, Kawasaki, Kanagawa, Japan; 3 College of Veterinary Medicine and Institute of Veterinary Science, Kangwon National University, Chuncheon, Gangwon, Republic of Korea

**Keywords:** alopecia, androgenetic alopecia, companion animals, drug delivery systems, one health, pharmaceutical formulations

## Abstract

Hair loss (alopecia) is a multifactorial condition affecting humans and companion animals, including dogs and cats, with etiologies involving hormonal, immune-mediated, genetic, infectious, allergic, parasitic, and behavioral mechanisms. Although androgenetic alopecia and alopecia areata are well characterized in humans, alopecia in veterinary patients more frequently reflects an underlying endocrine, hereditary, infectious, allergic, parasitic, or self-induced disorder. This review provides a comparative analysis of the pathophysiology, clinical presentation, pharmacological management, and pharmaceutical formulation of nonscarring alopecia across species. In humans, United States Food and Drug Administration-approved treatments, including topical minoxidil, oral finasteride, and selected Janus kinase inhibitors, are supplemented by emerging approaches such as platelet-rich plasma, low-level laser therapy, regenerative medicine, and nanotechnology-based delivery systems. In contrast, veterinary management primarily depends on identification and correction of the underlying disorder, with limited species-specific evidence and few dedicated formulations. Differences in follicular architecture, hair-cycle kinetics, skin permeability, drug metabolism, coat characteristics, and grooming behavior preclude direct extrapolation of human therapies to dogs and cats. Dermatophytosis further illustrates the One Health relevance of comparative alopecia management because successful control may require coordinated treatment of affected animals and humans together with environmental decontamination. This review highlights the need for species-specific clinical trials, safety assessment, and formulation strategies to support the development of effective and practical treatments for human and veterinary alopecia.

## 1 Introduction

Alopecia refers to the partial or complete loss of hair from normally hair-bearing skin. In medical terms, alopecia is broadly categorized into the non-scarring (non-inflammatory) and scarring (inflammatory) types, depending on whether permanent damage to the hair follicles occurs. The most common forms in humans include androgenetic alopecia (AGA) and alopecia areata (AA), each of which has distinct pathophysiological mechanisms. AGA is a progressive, non-scarring condition driven by an interplay between genetic predisposition and androgen metabolism, particularly dihydrotestosterone (DHT), which acts on hair follicle androgen receptors, leading to follicular miniaturization and shortening of the anagen (growth) phase (1). In contrast, AA is a T cell-mediated autoimmune disease that targets the hair follicles, resulting in patchy hair loss and varying degrees of regrowth (2).

In companion animals, particularly dogs and cats, alopecia is also a common dermatological complaint, often reflecting underlying endocrine, genetic, autoimmune, or infectious processes. Dogs can present with hormonally driven alopecia, such as hypothyroidism or hyperadrenocorticism-related hair loss; breed-specific syndromes, such as color dilution alopecia or follicular dysplasia; and idiopathic forms, such as alopecia X, which is especially prevalent in Pomeranians and Nordic breeds (3–5). Cats develop alopecia secondary to dermatophytosis, flea allergy dermatitis, psychogenic overgrooming, and systemic illness (6–8). Despite its prevalence, the mechanisms underlying alopecia in animals remain less thoroughly elucidated than those in humans.

The clinical significance of alopecia extends beyond cosmetic concerns. In humans, hair loss affects self-esteem, body image, and quality of life, with psychological effects sometimes comparable to those observed in patients with severe chronic illnesses (9). In veterinary patients, alopecia may indicate systemic disease, compromise skin barrier integrity, predispose patients to secondary infections, and reduce the perceived value of show and breeding animals, imposing medical and economic burdens on owners (10, 11).

The social and economic impacts of alopecia have driven considerable advances in therapeutic approaches, particularly in humans. Two pharmacological agents, topical minoxidil and oral finasteride, are United States Food and Drug Administration (FDA)-approved for treating AGA, with additional off-label and emerging therapies including low-level laser therapy (LLLT), platelet-rich plasma (PRP), microneedling, and nanotechnology-based drug delivery systems still under investigation (12). In contrast, veterinary medicine relies largely on empirical treatments, hormonal modulation, and the management of underlying diseases, with very few species-specific therapeutic agents or formulations being available (3, 13, 14).

Comparative research on the pharmacological and pharmaceutical management of alopecia across species is crucial for several reasons. First, fundamental differences exist among humans, dogs, and cats in terms of skin structure, follicular cycling, androgen metabolism, and drug absorption, distribution, metabolism, and excretion (ADME), all of which affect therapeutic efficacy and safety. Second, human-approved therapies cannot be indiscriminately applied to veterinary patients because of species-specific toxicities. For instance, topical minoxidil can cause severe cardiotoxicity in cats (15). Third, veterinary patients present unique challenges in drug delivery, such as grooming behaviors that limit the retention of topical products and owner compliance issues with long-term oral therapies (16). Although alopecia is broadly classified into scarring and nonscarring forms, this review focuses specifically on nonscarring alopecia because the pharmacological management and drug delivery strategies for scarring alopecias differ fundamentally from those for nonscarring alopecias. Accordingly, this review adopts a comparative approach by emphasizing the similarities and differences in hair biology, disease mechanisms, pharmacokinetics, and therapeutic responses among humans, dogs, and cats to facilitate the development of species-appropriate therapeutic strategies.

Therefore, the objective of this review was to: (i) provide a comprehensive overview of the pathophysiology and clinical presentation of alopecia in humans, dogs, and cats; (ii) analyze the mechanisms of action, efficacy, and safety of current and emerging pharmacological therapies; (iii) evaluate the design and suitability of pharmaceutical formulations and delivery systems across species; and (iv) identify challenges and opportunities in developing innovative, species-appropriate treatments. By synthesizing the current knowledge in a comparative context, a further aim of this review was to identify future research directions and facilitate the development of safe, effective, and patient-friendly therapeutics for alopecia in humans and companion animals.

## 2 Pathophysiology and types of alopecia in humans, dogs, and cats

Alopecia or hair loss arises from disruption of the normal hair cycle, which consists of the anagen (growth), catagen (regression), telogen (resting), and exogen (shedding) phases. Intrinsic factors such as hormonal and immune-mediated dysregulation and extrinsic factors such as infection and trauma can alter this cycle, leading to follicular atrophy or dysfunction. Although alopecia manifests across species, its underlying pathophysiological mechanisms and clinical classifications vary considerably between humans and companion animals.

### 2.1 Human alopecia: androgenetic, female pattern, and alopecia areata

AGA is the most prevalent form of hair loss in humans, affecting up to 50% of men and 40% of women by the age of 50 years (17). AGA is a genetically predisposed, progressive condition characterized by the miniaturization of hair follicles and shortening of the anagen phase. In men, the condition is androgen-dependent, with DHT, which is synthesized from testosterone by 5α-reductase, binding to androgen receptors in dermal papilla cells, triggering the expression of genes that inhibit hair follicle proliferation and induce miniaturization (18). In women, AGA is less strongly associated with androgens; it often presents as diffuse thinning over the crown with sparing of the frontal hairline, termed female pattern hair loss (FPHL) (19).

In contrast, AA is a non-scarring autoimmune condition that typically results in sharply demarcated, round patches of hair loss. AA involves the collapse of immune privilege at the hair follicle, allowing autoreactive CD8+ T cells to attack keratinocytes in the bulb region (20). Cytokines such as IFN-γ and IL-15 are crucial in mediating this inflammatory process (21–23).

### 2.2 Canine and feline alopecia: endocrine, infectious, genetic, and behavioral disorders

In dogs and cats, alopecia is also a common dermatological finding, but its etiologies are more diverse and are often species- or breed-specific (13, 24). Endocrinopathies are among the major causes of nonscarring alopecia in dogs (3, 13). Canine hypothyroidism commonly produces a dry, dull, or brittle hair coat, failure of hair regrowth after clipping, increased telogen retention, and bilaterally symmetrical, usually nonpruritic alopecia affecting the trunk and areas of friction while typically sparing the head and distal extremities (25, 26). Secondary seborrhea and recurrent bacterial or *Malassezia* dermatitis may also occur because thyroid hormone deficiency impairs epidermal turnover and cutaneous immune function (3, 25). In humans, thyroid dysfunction more commonly causes diffuse scalp hair thinning, reduced hair density, or changes in hair texture rather than the characteristic bilaterally symmetrical truncal alopecia and clipping-associated failure of hair regrowth observed in dogs (25, 27).

Hyperadrenocorticism also produces species-specific dermatological manifestations. In humans, Cushing's syndrome is characterized by skin atrophy, easy bruising, and violaceous striae, and may also be accompanied by scalp hair thinning as a consequence of chronic glucocorticoid excess (28, 29). In contrast, dogs with hyperadrenocorticism typically develop bilaterally symmetrical truncal alopecia, follicular atrophy, delayed hair regrowth, and skin atrophy. Additional dermatological abnormalities include comedones, delayed wound healing, recurrent bacterial skin infections, and calcinosis cutis in some affected animals (3, 30). These findings reflect chronic glucocorticoid-induced follicular and dermal atrophy and differ substantially from the clinical presentation observed in humans (3, 28).

Breed-associated disorders such as alopecia X, which occurs predominantly in Pomeranians and other Nordic breeds, are characterized by hair-cycle arrest (31, 32). Although its precise pathogenesis remains incompletely understood, altered steroid hormone metabolism, abnormal local follicular hormone metabolism, and failure of anagen initiation have all been proposed as contributing mechanisms (31, 32). Unlike human androgenetic alopecia, alopecia X is not considered an androgen-dependent follicular miniaturization disorder (31, 33).

Hereditary and structural hair disorders also represent important causes of alopecia in dogs. Color dilution alopecia is associated with dilute coat-color genotypes and abnormal melanin distribution within the hair shaft, resulting in hair fragility, follicular abnormalities, and progressive hypotrichosis or alopecia (4, 34). Follicular dysplasias comprise a heterogeneous group of breed-associated disorders characterized by abnormal formation of the hair shaft or follicle, often leading to recurrent hair breakage and poor coat quality (13, 35). These disorders differ fundamentally from human androgenetic alopecia because the primary abnormality involves genetically determined follicular or hair-shaft defects rather than androgen-mediated follicular miniaturization (13, 33).

Dermatophytosis is an important infectious cause of alopecia in dogs and cats and has direct comparative relevance to human tinea capitis because both conditions involve colonization and invasion of keratinized hair shafts by dermatophytes (36, 37). *Microsporum canis* is the predominant dermatophyte associated with feline dermatophytosis and is also frequently isolated from infected dogs, whereas other organisms, including members of the *Trichophyton mentagrophytes* complex and geophilic *Nannizzia* species formerly classified within the *Microsporum gypseum* complex, occur less consistently (36, 38). Young animals, long-haired cats, animals living in crowded or stressful environments, and those with concurrent disease or impaired immunity are at increased risk (36, 38).

Dermatophytes produce keratinolytic enzymes that permit colonization of the stratum corneum and hair shaft. Fungal hyphae and arthroconidia develop around or within infected hairs, weakening the shaft and causing breakage near the skin surface (36, 39). Consequently, affected dogs and cats may develop focal, multifocal, or generalized alopecia accompanied by scaling, crusting, follicular papules, erythema, or variable pruritus. Cats may remain minimally symptomatic or act as subclinical carriers, which is epidemiologically important because clinically normal animals can contribute to environmental contamination and zoonotic transmission (36, 38).

Human tinea capitis shares the same fundamental mechanism of dermatophyte invasion of scalp hairs but differs from canine and feline dermatophytosis in its epidemiology, predominant pathogens, and clinical expression (36, 37). Tinea capitis occurs primarily in children and may present as noninflammatory scaling with broken hairs, black-dot alopecia, diffuse scaling, or a highly inflammatory kerion (37, 40). Anthropophilic species such as *Trichophyton tonsurans* are important causes of person-to-person transmission in many regions, whereas zoophilic *M. canis* infection is commonly acquired following exposure to infected cats or dogs and often induces a more pronounced inflammatory response (36, 37). Thus, canine and feline dermatophytosis and human tinea capitis represent related hair-shaft infections, but their dominant reservoirs, transmission dynamics, and inflammatory patterns differ (36, 37).

Zoonotic transmission occurs through direct contact with infected or carrier animals and indirectly through contaminated hairs, bedding, grooming equipment, cages, furniture, clothing, transport carriers, and household surfaces. Arthroconidia attached to shed hairs may persist in the environment and serve as an ongoing source of reinfection (36, 39). Children, older adults, immunocompromised individuals, animal-care personnel, shelter workers, and members of multi-pet households are at increased risk. Therefore, treatment of the clinically affected animal alone may be insufficient when asymptomatic carrier animals or contaminated fomites remain within the household (36, 38).

Diagnosis in dogs and cats should integrate clinical examination with direct microscopic examination of affected hairs, Wood's lamp evaluation where appropriate, fungal culture, and dermatophyte PCR (36). Wood's lamp fluorescence is particularly useful for detecting hairs infected with certain strains of *M. canis*, although a negative result does not exclude dermatophytosis (36, 41). The principal therapeutic strategy in companion animals is to combine systemic antifungal therapy, which eradicates infection within the follicle and developing hair shaft, with topical therapy to reduce surface spores, coat contamination, and zoonotic transmission (36, 42). Itraconazole or terbinafine is commonly used for systemic treatment, whereas lime sulfur rinses, enilconazole where available, or antifungal shampoos may be used as adjunctive topical therapy. Environmental decontamination, removal of infected hairs, laundering of washable materials, cleaning of contaminated surfaces, and evaluation of in-contact animals are essential components of disease control (36, 42).

Systemic antifungal therapy is likewise required for human tinea capitis because topical agents alone cannot adequately penetrate infected hair follicles or hair shafts (37, 43). However, the choice of antifungal agent depends on the causative organism, regional epidemiology, patient age, and safety considerations. Adjunctive antifungal shampoos may reduce fungal shedding and transmission but are not curative when used alone (37, 40). From a One Health perspective, successful management of zoonotic *M. canis* infection requires coordinated treatment of affected humans and animals, identification and management of asymptomatic animal carriers when appropriate, and environmental control to interrupt recurrent interspecies transmission (36, 37).

In cats, alopecia is frequently caused by self-induced hair removal secondary to pruritic skin diseases, including flea allergy dermatitis, food hypersensitivity, atopic skin disease, and ectoparasitic infestations. Consequently, apparent feline overgrooming should not be considered psychogenic until infectious, parasitic, allergic, and other underlying medical conditions have been excluded (8, 44). True psychogenic overgrooming is associated with stress, anxiety, environmental conflict, or compulsive behavior and typically results in symmetrical areas of broken hair on the abdomen, flanks, or limbs (7, 45). This condition provides a useful comparison with human trichotillomania. Whereas trichotillomania is characterized by recurrent hair pulling, feline psychogenic overgrooming results primarily from excessive licking or chewing. Despite these behavioral differences, both disorders produce self-induced alopecia and often require combined behavioral, environmental, and pharmacological management (7, 46). Endocrine alopecia is considerably less common in cats than in dogs, although feline hyperadrenocorticism may occasionally be associated with poor coat quality, alopecia, and severe skin fragility (47, 48).

Collectively, these disorders demonstrate that alopecia in dogs and cats arises from a broader and more species-specific spectrum of endocrine, hereditary, infectious, allergic, parasitic, and behavioral mechanisms than the predominantly androgen-mediated and autoimmune disorders emphasized in human clinical practice (13, 33). These etiological differences must be interpreted together with species-specific follicular biology, skin characteristics, drug metabolism, and grooming behavior, as discussed in the following section.

### 2.3 Comparative hair follicle biology and its pharmacological implications in humans, dogs, and cats

**Figure 1:**
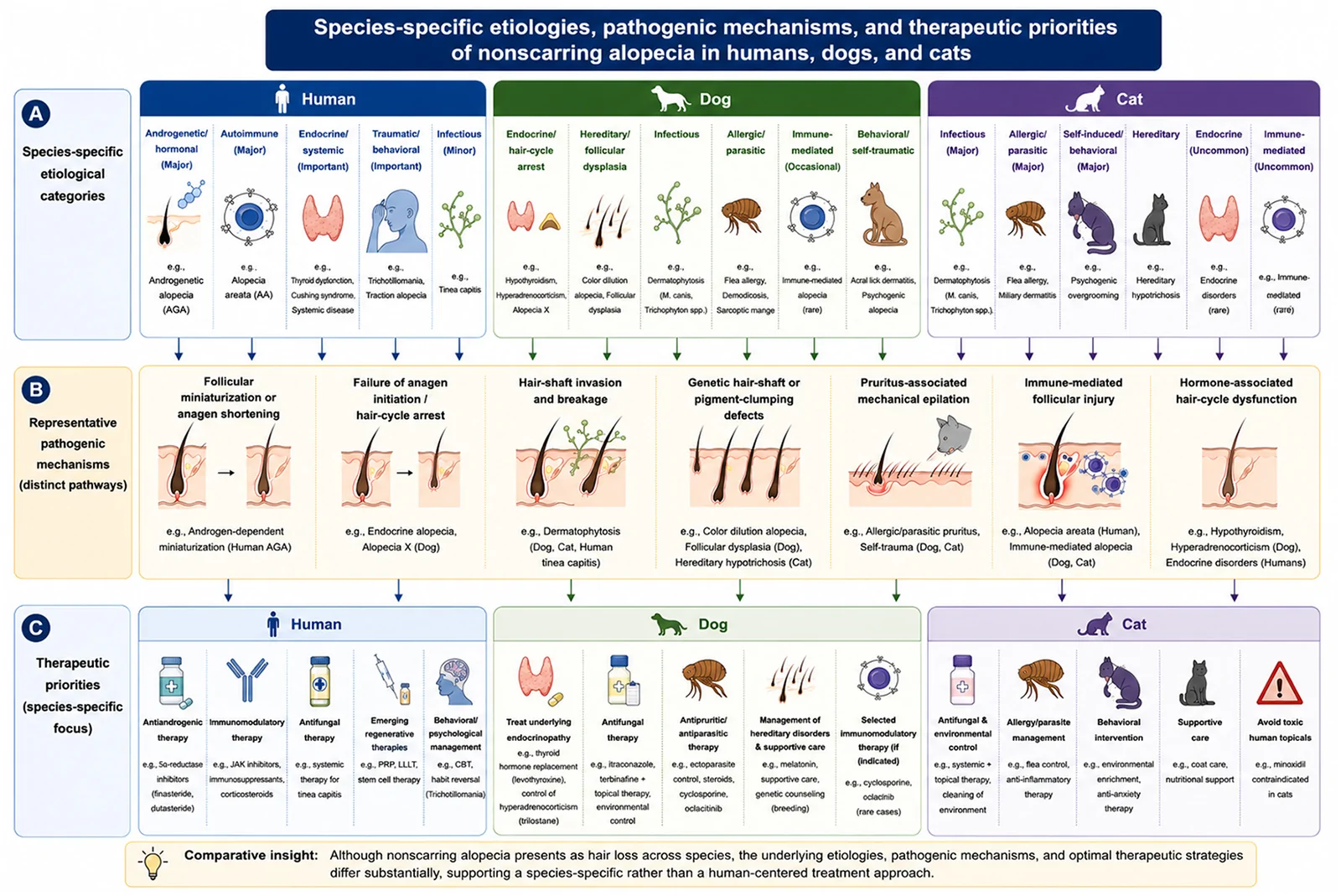
Species-specific etiologies, representative pathogenic mechanisms, and therapeutic priorities in nonscarring alopecia in humans, dogs, and cats. Human nonscarring alopecia is primarily characterized by androgenetic alopecia and alopecia areata, with endocrine/systemic disorders, traumatic or behavioral alopecia, and infectious causes representing less common etiologies. In dogs, endocrine and hair-cycle disorders, hereditary follicular dysplasias, dermatophytosis, allergic or parasitic diseases, and occasional immune-mediated or behavioral alopecias constitute the major disease categories. In cats, dermatophytosis, allergic or parasitic dermatitis, and self-induced overgrooming are the predominant causes of alopecia, whereas endocrine and immune-mediated disorders are comparatively uncommon. These diverse etiologies result in hair loss through distinct pathogenic mechanisms, including follicular miniaturization, failure of anagen initiation or hair-cycle arrest, hair-shaft invasion and breakage, genetic hair-shaft or pigment abnormalities, pruritus-associated mechanical epilation, immune-mediated follicular injury, and hormone-associated hair-cycle dysfunction. Consequently, therapeutic management should be tailored to the underlying species-specific disease and may include antiandrogenic or immunomodulatory therapy, treatment of endocrinopathies, antifungal or antiparasitic therapy, behavioral intervention, supportive management, and species-appropriate pharmaceutical formulations while avoiding the inappropriate use of human topical medications in companion animals.

Hair follicles in humans, dogs, and cats share a conserved basic organization consisting of an epithelial component, including the outer and inner root sheaths and hair matrix, and a mesenchymal dermal papilla that regulates follicular growth and cycling. In all three species, epithelial stem cells located primarily within the bulge region contribute to cyclic follicular regeneration, whereas reciprocal signaling between the dermal papilla, matrix keratinocytes, perifollicular vasculature, immune cells, and the surrounding extracellular matrix determines entry into and maintenance of the anagen phase (49, 50). Nevertheless, substantial interspecies differences exist in follicular architecture. Human scalp follicles generally occur as individual pilosebaceous units and produce relatively long terminal hairs. In contrast, canine and feline follicles are commonly organized as compound follicular units in which a primary follicle is accompanied by multiple secondary follicles (49, 50). Dogs and cats also have a higher follicular density and a more heterogeneous combination of primary guard hairs and secondary undercoat hairs (49, 50). These structural characteristics complicate the uniform application of topical products through the hair coat and may alter the distribution and retention of drugs within follicular openings (51, 52). Figure 1 summarizes the species-specific etiological categories, representative pathogenic mechanisms, and therapeutic priorities of nonscarring alopecia in humans, dogs, and cats.

Hair-cycle kinetics also differ markedly among species. Human scalp follicles display asynchronous cycling and a prolonged anagen phase that may persist for several years, followed by relatively short catagen and telogen phases (53, 54). This prolonged growth phase permits the production of long scalp hair and contributes to the gradual clinical progression of androgenetic alopecia (33, 53). In dogs, anagen duration and the proportion of follicles in telogen vary considerably according to breed, coat phenotype, body region, season, age, and endocrine status (13, 51). Many canine follicles remain in telogen for extended periods, and endocrine disorders or primary hair-cycle abnormalities may prevent appropriate anagen re-entry (13, 51). Feline hair cycling is likewise influenced by body region and seasonal photoperiod, although quantitative data remain much more limited than those available for humans and dogs (51, 55). Consequently, treatment-response intervals established for human alopecia cannot be directly extrapolated to veterinary patients, and clinical trials in dogs and cats should account for species-, breed-, and season-dependent coat cycling (13, 51).

Several major regulatory pathways are conserved across mammalian hair follicles, including Wnt/β-catenin, Sonic hedgehog, bone morphogenetic protein, fibroblast growth factor, transforming growth factor-β, and vascular endothelial growth factor signaling (51, 56, 57). These pathways regulate follicular stem-cell activation, matrix-cell proliferation, dermal papilla activity, angiogenesis, and the transition between anagen, catagen, and telogen (57–59). However, their relative clinical importance differs among species and alopecia subtypes. In human androgenetic alopecia, androgen receptor signaling and local conversion of testosterone to dihydrotestosterone within susceptible scalp follicles induce progressive miniaturization and shortening of anagen (60, 61). Comparable androgen-dependent follicular miniaturization has not been established as a major mechanism in most canine or feline alopecias (13, 60). Instead, endocrine disruption, genetic hair-shaft abnormalities, infectious injury, allergic or parasitic pruritus, and self-induced hair removal represent major causes of alopecia in veterinary patients (13, 62, 63). Likewise, collapse of hair-follicle immune privilege is central to human alopecia areata and has a recognized canine counterpart, but naturally occurring immune-mediated alopecia is uncommon in cats (13, 20, 51).

Endocrine regulation also differs among species. Thyroid hormone contributes to normal follicular metabolism and anagen maintenance; therefore, canine hypothyroidism commonly produces delayed anagen initiation, increased telogen retention, poor hair regrowth, and bilaterally symmetrical truncal alopecia (13, 64). Chronic glucocorticoid excess suppresses follicular activity and protein synthesis and produces follicular atrophy, comedones, thin skin, and symmetrical truncal alopecia in dogs (3, 13). In cats, spontaneous hyperadrenocorticism is uncommon and is more often associated with fragile skin, poor coat quality, alopecia, and concurrent insulin-resistant diabetes mellitus (47, 48). By contrast, thyroid dysfunction and Cushing syndrome in humans can cause diffuse scalp hair thinning but do not usually reproduce the characteristic breed- and trunk-associated patterns observed in dogs (27, 65).

Interspecies pharmacokinetic differences further limit direct therapeutic extrapolation. Canine and feline skin differs from human skin in thickness, lipid composition, follicular density, and regional permeability, and topical drug delivery is further influenced by the presence of dense hair coats (66, 67). Grooming may remove a portion of the applied dose, reduce local follicular exposure, and increase oral exposure through licking (68, 69). Cats also have limited glucuronidation capacity for several substrates, which contributes to their heightened susceptibility to certain drugs and xenobiotics (70, 71). These characteristics are clinically relevant to alopecia treatment because a topical formulation that is effective and well tolerated in humans may produce inadequate local delivery, excessive systemic exposure, or severe toxicity in a companion animal (66, 70). Minoxidil is a prominent example: it is routinely used for human androgenetic alopecia but can cause life-threatening cardiovascular toxicosis in cats following dermal or oral exposure (72).

Accordingly, similarities in basic follicular organization do not imply therapeutic interchangeability. Human alopecia treatment frequently targets androgen signaling, follicular vascular support, or autoimmune pathways, whereas treatment in dogs and cats must more often address the underlying endocrine, infectious, allergic, parasitic, genetic, or behavioral disorder (13, 73). The major types, etiologies, clinical patterns, and diagnostic considerations of alopecia in humans, dogs, and cats are summarized in Table 1. Species-specific differences in follicular structure, hair-cycle kinetics, molecular regulation, skin permeability, drug metabolism, grooming behavior, and predominant alopecia mechanisms are summarized in Table 2. Representative pharmacological and adjunctive therapies, together with their evidence status and species-specific safety considerations, are presented in Table 3. These differences determine not only the selection of pharmacological targets but also the required treatment duration, dosage form, safety evaluation, and route of administration.

**Table 1:** Major alopecia types and etiologies among humans, dogs, and cats (overview).

Species	Common alopecia types	Common etiologies	Typical clinical pattern	Key diagnostics/differentials
Humans	AGA; female pattern hair loss FPHL; AA.	AGA/FPHL: genetic predisposition + androgen/DHT effects on susceptible scalp follicles. AA: autoimmune (collapse of follicular immune privilege; CD8+ T cell response).	AGA: bitemporal recession/vertex thinning in men; FPHL: diffuse crown thinning with frontal hairline sparing. AA: well-demarcated patches; may relapse.	History + pattern recognition; dermoscopy/trichoscopy; hair pull test; labs as indicated (e.g., thyroid/iron); biopsy if atypical/scarring suspected.
Dogs	Endocrine alopecias; noninflammatory alopecia syndromes (e.g., alopecia X); breed-associated follicular disorders (color dilution alopecia, follicular dysplasia); autoimmune AA-like alopecia.	Hypothyroidism; hyperadrenocorticism; idiopathic hair cycle arrest; genetic hair shaft/follicle defects; less commonly immune-mediated or infectious causes.	Often bilaterally symmetrical truncal alopecia with hyperpigmentation; nonpruritic in many endocrine/idiopathic cases. Coat quality changes; delayed regrowth after clipping (endocrine).	Rule out parasites/infection first; endocrine testing (thyroid panel, ACTH stimulation/LDDS); skin cytology; fungal culture; biopsy for noninflammatory alopecia classification.
Cats	Secondary alopecia most common: dermatophytosis; flea allergy dermatitis/other pruritic dermatoses; demodicosis; psychogenic overgrooming; systemic illness; endocrine alopecia uncommon.	Ectoparasites and hypersensitivity; fungal infection; grooming/behavioral causes; occasional endocrine/neoplastic associations.	Frequently self-induced alopecia with broken hairs; often pruritic; may be multifocal (abdomen/flanks) depending on cause.	Flea combing and ectoparasite evaluation; skin scraping/tape tests; fungal culture/PCR; assess pruritus and behavior; consider systemic work-up if generalized.

AGA, androgenetic alopecia; FPHL, female pattern hair loss; AA, alopecia areata; DHT, dihydrotestosterone; ACTH, adrenocorticotropic hormone; LDDS, low-dose dexamethasone-suppression test; PCR, polymerase chain reaction.

**Table 2:** Comparative hair follicle biology and pharmacological implications in humans, dogs, and cats.

Comparative parameter	Humans	Dogs	Cats	Pharmacological or clinical implication
Follicular organization	Most scalp follicles occur as individual pilosebaceous units producing terminal hairs.	Compound follicles are common; one primary follicle is associated with multiple secondary follicles producing guard and undercoat hairs.	Compound follicles and a dense mixture of primary and secondary hairs are common.	Dense compound follicular units and fur may impede uniform topical application in dogs and cats despite a high number of follicular openings.
Hair-coat characteristics	Relatively uniform terminal scalp hairs with minimal self-removal behavior.	Marked breed-related variation in coat length, density, undercoat, and shedding pattern.	Dense coat, regional variation, and frequent grooming substantially influence apparent hair loss and topical exposure.	Veterinary efficacy studies must account for breed, body region, seasonal shedding, and grooming.
Hair-cycle pattern	Asynchronous cycling; scalp anagen commonly lasts several years.	Anagen and telogen duration vary by breed, coat type, body region, season, and endocrine status; prolonged telogen is common in several disorders.	Cycling varies by body region, breed, photoperiod, and season; quantitative information is relatively limited.	Human treatment timelines cannot be directly transferred to dogs or cats; veterinary trials may require breed- and season-specific observation periods.
Follicular stem-cell and dermal papilla regulation	Bulge stem cells and dermal papilla signaling are well characterized.	Conserved bulge and dermal papilla compartments are present, including stem-cell populations within compound follicles.	The same fundamental compartments are present, but species-specific molecular characterization is less extensive.	Conserved regenerative pathways provide translational targets, but dosing and efficacy require species-specific validation.
Major molecular pathways	Wnt/β-catenin, Sonic hedgehog, BMP, FGF, TGF-β, VEGF, androgen receptor, and immune-privilege pathways are well characterized.	Core cycling pathways are conserved, but endocrine status, breed-associated genetics, and hair-cycle arrest have greater clinical relevance than androgen-driven miniaturization.	Core pathways are presumed to be conserved; infectious, allergic, parasitic, and behavioral factors are more clinically prominent.	Shared pathways may support translational research, but the dominant pathogenic target differs by alopecia subtype and species.
Androgen responsiveness	DHT–androgen receptor signaling is central to androgenetic alopecia in susceptible scalp follicles.	The role of androgen signaling is inconsistent and is not established as the primary mechanism in most canine alopecias.	A human-like androgenetic alopecia syndrome is not well established.	Finasteride and related antiandrogens should not be extrapolated from human AGA without veterinary efficacy and reproductive-safety data.
Immune privilege	Collapse of follicular immune privilege is central to alopecia areata.	A naturally occurring canine counterpart of alopecia areata has been documented.	Immune-mediated follicular alopecia is uncommon and poorly characterized.	JAK inhibitors or immunosuppressive therapies may have comparative relevance, but veterinary evidence remains limited.
Endocrine regulation	Thyroid dysfunction and glucocorticoid excess may cause diffuse hair thinning.	Hypothyroidism and hyperadrenocorticism are major causes of bilaterally symmetrical, nonpruritic alopecia and delayed regrowth.	Endocrine alopecia is uncommon; hyperadrenocorticism may produce alopecia, poor coat, and severe skin fragility.	Veterinary treatment should correct the underlying endocrinopathy rather than directly stimulate hair growth.
Skin barrier and topical absorption	Relatively thick barrier; topical dosing and cosmetic acceptability are major considerations.	Skin thickness and permeability differ from human skin and vary by body site and breed.	Dermal exposure is combined with a high probability of oral exposure through grooming.	Topical concentrations, vehicles, and exposure limits must be independently established for each species.
Drug metabolism	Human hepatic metabolic pathways form the reference basis for most alopecia drugs.	Drug clearance may differ from humans and varies according to drug and breed.	Reduced glucuronidation capacity for several substrates increases sensitivity to selected xenobiotics.	Human doses cannot be converted solely by body weight; feline metabolic limitations require particular caution.
Grooming and licking	Generally not relevant to scalp drug retention.	Licking may remove topically applied drugs and cause secondary oral exposure.	Frequent grooming produces substantial risk of dose removal, oral ingestion, and household cross-exposure.	Rapid-drying, low-residue, non-toxic, or non-topical formulations are preferable for many veterinary applications.
Predominant alopecia mechanisms	Androgenetic, autoimmune, inflammatory, infectious, or treatment-related.	Endocrine, hereditary or breed-associated, infectious, allergic/parasitic, immune-mediated, and hair-cycle disorders.	Infectious, allergic/parasitic, self-induced or behavioral, and less commonly endocrine or immune-mediated.	Comparative figures and treatment frameworks must represent the full veterinary etiologic spectrum rather than focusing primarily on human AGA and AA.

**Table 3:** Representative alopecia therapies: evidence status and key safety considerations by species.

Therapy/agent	Humans (typical use)	Dogs (typical use)	Cats (typical use)	Key safety notes
Topical minoxidil	AGA/FPHL; long-term maintenance required.	Rarely used; limited validation; consider risk of systemic absorption.	Avoid/contraindicated due to severe toxicosis risk.	Cats: dermal exposure can be fatal; prevent household cross-exposure (hands, bedding).
Finasteride/dutasteride	Male AGA (finasteride FDA-approved); dutasteride off-label.	Studied for BPH; alopecia efficacy unproven; reproductive effects reported.	Not established for common feline alopecias.	Teratogenic risk in pregnancy (humans); consider handling precautions.
Spironolactone (antiandrogen)	Off-label for FPHL/hyperandrogen states.	Not standard for canine alopecia; hormonal manipulation depends on diagnosis.	Not standard; most feline alopecia is secondary (parasite/behavior/infection).	Monitor electrolytes and blood pressure in humans; species-specific veterinary use requires caution.
Corticosteroids (local/systemic)	AA (intralesional), inflammatory/scarring disorders as indicated.	Immune-mediated alopecias/dermatoses (case-by-case).	Immune-mediated dermatoses/pruritus as indicated; avoid if infectious causes uncontrolled.	Chronic use: metabolic and infection risks; monitor appropriately.
Cyclosporine and other immunosuppressants	Selected inflammatory alopecias (less common); specialist use.	Autoimmune dermatopathies/AA-like disease; limited evidence.	Immune-mediated dermatoses; individualized.	Drug interactions and monitoring (renal/hepatic, infections) are important.
Levothyroxine	Treat hypothyroidism-associated hair changes when present.	First-line for canine hypothyroidism-related alopecia.	Rare indication; use only with confirmed hypothyroidism (uncommon).	Avoid empiric therapy without diagnosis; monitor clinical response and T4.
Trilostane/mitotane	Cushing-related changes managed medically/surgically in humans.	Core therapy for canine hyperadrenocorticism with alopecia.	Rare; specialist management if confirmed.	Requires endocrine monitoring; risk of hypoadrenocorticism if overtreated.
Melatonin	Sometimes used off-label; evidence heterogeneous.	Used for alopecia X/seasonal flank alopecia; mixed evidence.	Occasional use; variable PK and response.	Inter-individual variability; sedation possible; assess comorbidities.
PRP (intradermal)	Adjunct for AGA/other hair loss; RCTs support benefit.	Limited dermatology-specific evidence; used in other veterinary indications.	Not systematically studied for feline alopecia.	Standardization of preparation and dosing varies widely.
LLLT	Device-based adjunct; evidence supports increased hair density in some studies.	Pilot study suggests potential in noninflammatory alopecia.	Dermatologic use reported, but alopecia-specific evidence limited.	Device parameters and treatment schedule affect outcomes.

AGA, androgenetic alopecia; FPHL, female pattern hair loss; AA, alopecia areata; FDA, United States Food and Drug Administration; BPH, benign prostatic hyperplasia; PRP, platelet-rich plasma; RCTs, randomized controlled trials; LLLT, low-level laser therapy; PK, pharmacokinetics.

## 3 Pharmacological treatments for alopecia and their mechanisms of action

The pharmacological management of alopecia focuses on the counteraction of the molecular and cellular mechanisms that impair normal hair follicle function. Therapeutic strategies aim to restore hair growth by suppressing androgen-mediated follicular miniaturization, enhancing vascular support for the follicle, and prolonging the anagen phase (74, 75). Although several agents are approved and routinely used in humans, veterinary medicine still relies primarily on empirical and off-label therapies, reflecting a lack of species-specific data and inherent biological differences in hair follicle physiology.

In humans, the two drugs officially approved by the FDA for the treatment of AGA are topical minoxidil and oral finasteride (76). Minoxidil was originally developed as an antihypertensive agent and later found to stimulate hair growth (77). Its precise mechanism of action remains incompletely understood; however, evidence suggests that it opens adenosine triphosphate (ATP)-sensitive potassium channels in the dermal papilla cells, promotes vascular endothelial growth factor (VEGF) expression, improves blood flow around the follicle, and prolongs the anagen phase. These effects help sustain follicle viability and counteract miniaturization. Finasteride, on the other hand, is a selective inhibitor of type II 5α-reductase, the enzyme responsible for converting testosterone into DHT (78). DHT binds to androgen receptors within the follicle and initiates gene expression programs that lead to miniaturization and early transition to telogen; however, finasteride reduces scalp DHT levels by approximately 60%, thereby slowing or reversing follicular atrophy in men. In women, its use is limited because of the risk of teratogenicity (79). Another oral 5α-reductase inhibitor, dutasteride, which blocks the type I and type II isoenzymes of 5α-reductase, has shown even greater efficacy in clinical trials, although it remains off-label (80). Additionally, spironolactone, an aldosterone antagonist with anti-androgenic properties, is widely prescribed off-label for women with FPHL (81). This agent blocks androgen receptors and inhibits androgen synthesis, thereby reducing follicular exposure to androgens. More recently, newer therapies such as platelet-rich plasma (PRP), Janus kinase (JAK) inhibitors—including the FDA-approved agents baricitinib and ritlecitinib for severe alopecia areata—low-level laser therapy, and agents targeting Wnt/β-catenin signaling have expanded the therapeutic landscape for human alopecia (82–85).

The biological pathways targeted by these therapies generally fall into three major categories. First, agents such as finasteride and dutasteride reduce DHT production and signaling, thereby mitigating androgen-driven follicular miniaturization (86). Second, agents such as minoxidil and VEGF analogs promote perifollicular vascularization and prolong the anagen phase, thereby fostering a microenvironment conducive to hair growth (57, 75, 87). Third, immunosuppressive or immune-modulating therapies, including JAK inhibitors and corticosteroids, suppress the autoimmune attacks underlying conditions such as AA (88, 89). Experimental approaches have also explored activation of Wnt/β-catenin signaling and stimulation of follicular stem cells as strategies for regenerating dormant or atrophic follicles (90, 91). A pathway-oriented comparison of the therapeutic targets and representative treatments across species is presented in Table 4.

**Table 4:** Therapeutic targets and pathway-level translation across species.

Target pathway	Humans: commonly used therapies	Dogs: translation/use	Cats: translation/use	Key notes
Androgen-DHT-androgen receptor signaling	Finasteride (FDA-approved for male AGA) and off-label dutasteride; antiandrogens for FPHL (e.g., spironolactone).	5-alpha-reductase inhibitors have PK data but clinical efficacy for canine alopecia is unvalidated; reproductive effects are a concern in males.	Not validated; antiandrogen strategies rarely indicated for common feline alopecias.	Central mechanism for AGA in humans; less clearly central for most canine/feline alopecias.
Anagen promotion/perifollicular vascular support	Topical minoxidil (FDA-approved for AGA/FPHL); adjuncts affecting growth factors (VEGF-related signaling).	Minoxidil rarely used due to limited protocols and safety concerns; focus often on treating underlying cause.	Minoxidil is contraindicated due to high toxicity risk from dermal exposure and grooming.	Formulation matters (solutions vs. foam; irritation differences).
Immune modulation (autoimmune AA)	Intralesional/topical/ systemic corticosteroids; FDA-approved JAK inhibitors (baricitinib and ritlecitinib) for severe alopecia areata.	Immune-mediated alopecias treated case-by-case with corticosteroids/ cyclosporine/other immunosuppressants; limited controlled evidence.	Immune-mediated dermatoses exist; treatment individualized; evidence for primary alopecia indications remains limited.	Risk-benefit and monitoring are critical with chronic immunosuppression.
Endocrine normalization/hormonal modulation (non-androgen)	Treat underlying endocrine disorders when present (e.g., thyroid disease, hypercortisolism).	Core strategy for common canine endocrine alopecias: levothyroxine for hypothyroidism; trilostane/mitotane for hyperadrenocorticism.	Endocrine causes are uncommon but should be considered; treat underlying disease when confirmed.	Often yields hair regrowth as hair cycle normalizes; may take weeks to months.
Behavioral/infectious/ ectoparasite drivers (esp. cats)	Less common primary drivers for scalp alopecia; treat underlying dermatoses if present.	Treat ectoparasites/infections if present; address pruritic dermatoses.	First-line for many feline cases: parasite control, antifungals for dermatophytosis, and behavior management for overgrooming.	Species behavior (grooming/licking) strongly shapes outcomes and topical feasibility.

AGA, androgenetic alopecia; FPHL, female pattern hair loss; AA, alopecia areata; DHT, dihydrotestosterone; VEGF, vascular endothelial growth factor; FDA, United States Food and Drug Administration; JAK, Janus kinase.

In contrast, evidence-based management of alopecia in dogs and cats remains largely dependent on the underlying cause, and the level of clinical evidence varies considerably among different therapeutic approaches. Few species-specific regulatory-approved pharmacological therapies are currently available for the treatment of primary alopecia in companion animals in many jurisdictions. Although minoxidil is theoretically capable of stimulating hair growth, it is contraindicated in cats because of its extreme toxicity in this species (15). Even small amounts can induce lethargy, pulmonary edema, and potentially fatal cardiovascular collapse. Minoxidil is rarely used in dogs due to safety concerns and the absence of established and validated treatment protocols (15). Instead, the mainstay of therapy for hormonally mediated alopecias, such as those caused by hypothyroidism or hyperadrenocorticism, involves appropriate endocrine management with levothyroxine for thyroid deficiency or trilostane to suppress adrenal hormone production (92, 93). In cases of sex hormone-related alopecia, castration often improves the coat by eliminating excess androgen or estrogen production. Additionally, melatonin supplementation has shown benefits in dogs with idiopathic or seasonal alopecia, likely due to its effects on hair cycle regulation (94). For autoimmune-mediated alopecias in dogs, such as those resembling AA, immunosuppressive agents such as corticosteroids, cyclosporine, and azathioprine may be used, although evidence supporting their efficacy is limited (95). In addition, recent veterinary studies have highlighted the potential of platelet-rich plasma (PRP) and mesenchymal stem cell therapies as regenerative approaches in canine medicine (96, 97). However, evidence supporting the efficacy of these regenerative therapies in canine alopecia remains limited, and further controlled clinical studies are required before they can be recommended for routine clinical use. In cats, alopecia is commonly secondary to underlying dermatological or behavioral problems, particularly psychogenic overgrooming; therefore, treatment is directed at identifying and removing the inciting cause rather than at the hair follicle itself (8).

Species-specific differences in drug responses and hair follicle biology further complicate the translation of human therapies into veterinary medicine. In humans, type II 5α-reductase is abundantly expressed in scalp hair follicles, particularly within the dermal papilla, making DHT a critical factor in the pathogenesis of AGA (98–100). Consequently, 5α-reductase inhibitors such as finasteride are effective in humans, whereas their clinical efficacy for alopecia in dogs and cats has not been adequately validated (101, 102). Similarly, the baseline proportion of hair follicles in the telogen phase is much higher in dogs than in humans, and hair cycle dynamics differ substantially across species, which may affect responses to anagen-prolonging agents (64, 103–105). Differences in pharmacokinetics also play an important role. In particular, cats exhibit unique hepatic metabolic pathways that make them vulnerable to adverse effects from drugs such as minoxidil, which are tolerated at much higher doses in humans and dogs (70).

Overall, although humans benefit from well-defined pharmacological options targeting specific pathways involved in hair follicle miniaturization, vascularization, and immune privilege, the treatment of alopecia in companion animals remains less developed. Veterinary patients rely primarily on supportive and empirical therapies tailored to the underlying etiology, reflecting not only gaps in clinical evidence but also intrinsic biological differences, while highlighting opportunities for species-adapted therapeutic development. The currently used pharmacological and adjunctive therapies for alopecia, together with their indications, evidence levels, and species-specific safety considerations, are summarized in Table 3.

## 4 Formulations and drug delivery strategies for alopecia treatments

The clinical efficacy of pharmacological agents for alopecia depends not only on their pharmacodynamics, but also on their formulation and delivery methods. Because hair follicles are small, dynamic structures embedded within the skin, effective therapy often requires overcoming the formidable barrier function of the stratum corneum to deliver drugs to the follicular niche (106). In addition, treatment adherence and safety considerations strongly affect formulation choices, which differ markedly between humans and companion animals owing to behavioral and physiological differences (107).

In humans, the two FDA-approved treatments for AGA, minoxidil and finasteride, use distinct delivery approaches (76). Minoxidil is administered topically, typically in the form of solutions, foams, or gels, which are designed to enhance skin absorption and target the perifollicular microenvironment while minimizing systemic exposure (108). Conventional solutions contain alcohol and propylene glycol as penetration enhancers, which can irritate some users (109). To improve tolerability and cosmetic acceptability, foam-based formulations with reduced propylene glycol content have been developed and shown to achieve comparable efficacy with improved patient satisfaction (110). Conversely, finasteride is delivered orally as a tablet because its target (5α-reductase) is located systemically in dermal papilla cells and the prostate (111, 112). Other experimental or adjunctive treatments for alopecia, such as PRP or botulinum toxin, are delivered directly into the scalp *via* intradermal injections (113, 114).

Emerging drug delivery technologies are designed to enhance follicular targeting and overcome the limitations of conventional drug formulations. For example, nanoparticles and liposomes have been investigated as carriers of minoxidil, finasteride, and other agents because they can penetrate deeper into the follicular orifices and release drugs in a controlled manner (115, 116). Hydrogels are additional formulations that can increase drug residence time and enable controlled local delivery, thereby limiting systemic exposure (117). Such advanced delivery systems not only improve pharmacokinetic profiles but also address cosmetic concerns, which are key factors in treatment adherence in humans (118, 119).

Formulation challenges are even more pronounced in veterinary medicine. Dogs and cats exhibit species-specific behaviors such as licking, grooming, and scratching, which interfere with the retention and absorption of topical products (120). Furthermore, their dense coats and differences in skin anatomy complicate uniform drug application and absorption (121). Oral medications are commonly used, but present difficulties, particularly in cats, which are notoriously resistant to pill administration (122). Additionally, the risk of systemic side effects limits the use of certain drugs, and the unique hepatic metabolism in cats increases their susceptibility to toxicity from agents such as minoxidil (70).

In addition, self-grooming and licking behaviors may result in unintended oral exposure following topical drug application. Consequently, part of the applied dose may undergo gastrointestinal absorption and first-pass hepatic metabolism, thereby reducing local drug availability while increasing the potential for systemic exposure. These challenges highlight the importance of species-specific formulation design. Recent veterinary pharmaceutical research has explored rapid-drying vehicles that shorten the period during which topical products remain accessible for licking, taste-masking technologies that discourage oral ingestion, and formulation strategies aimed at maximizing local skin retention while minimizing systemic exposure. Such approaches may improve therapeutic efficacy, safety, and owner compliance in companion animals (123, 124).

To address these challenges, species-specific and user-friendly formulations are urgently required in veterinary practice. One promising strategy involves the development of chewable tablets or flavored treats that improve compliance in dogs, and to some extent, cats (125, 126). In addition, pet-specific sprays, foams, or spot-on solutions that minimize residue and allow rapid drying have been explored to increase owner acceptance and reduce self-grooming after application. The encapsulation of drugs in liposomes has also been extensively investigated as a strategy to enhance follicular uptake and enable sustained local delivery, thereby reducing the need for frequent dosing (127, 128).

Differences in skin structure and physiology between humans and animals also impact formulation design. Canine and feline skin are thinner and have a higher density of hair follicles than human skin, which can increase percutaneous absorption but also increase the risk of local irritation or systemic toxicity (50, 129, 130). The pH, lipid composition, and turnover rate of the stratum corneum differ too and affect drug partitioning and retention in the follicular niche. These differences underscore the importance of tailoring penetration enhancers, vehicle compositions, and dosing regimens specifically to each species.

In summary, the effective treatment of alopecia requires not only potent pharmacological agents but also appropriately designed delivery systems that maximize follicular targeting, minimize systemic exposure, and improve patient or owner compliance. Advances in formulation technologies, including foams, nanoparticles, and hydrogels, have expanded strategies to enhance drug delivery and local tolerability in humans (131, 132). However, in companion animals, behavioral and physiological differences impose additional barriers that necessitate the development of innovative, species-appropriate formulations, including palatable oral dosage forms and safer, pet-friendly topical systems (133, 134). Future research on veterinary-specific delivery technologies holds promise for enhancing the safety and effectiveness of alopecia treatments in dogs and cats. A comparison of the formulation types and drug delivery strategies used in human and veterinary alopecia therapies is presented in Table 5.

**Table 5:** Formulations and drug-delivery strategies: human vs. veterinary considerations.

Delivery/ formulation	Human examples	Veterinary opportunities	Advantages	Limitations/ constraints
Topical solution/lotion	Minoxidil solutions with alcohol/propylene glycol.	Limited by coat and licking; may be used for localized dermatoses.	Simple, low-cost; scalable.	Irritation; variable penetration; high risk of ingestion in pets (especially cats).
Topical foam	5% minoxidil foam improves tolerability vs. solution.	Faster drying, less residue may improve owner acceptance (species-specific development needed).	Better cosmetic feel; potentially improved adherence.	Still vulnerable to grooming/licking; distribution through fur is difficult.
Oral tablets/capsules	Finasteride tablets; systemic action.	Useful for endocrine therapies (e.g., trilostane) but pill compliance can be poor in cats.	Precise dosing; systemic reach.	Owner compliance barriers; adverse systemic effects; requires monitoring.
Palatable chewables/flavored suspensions	Less common in humans for alopecia.	Chewables (dogs) and flavored liquids (cats) improve compliance.	Higher acceptance; easier long-term administration.	Formulation complexity; stability; taste masking; dose flexibility.
Intradermal injections (e.g., PRP)	PRP for hair regrowth.	Potentially adaptable for veterinary dermatology; evidence still limited.	Bypasses SC barrier; targets follicular niche.	Invasiveness; cost; standardization challenges.
Nanocarriers (liposomes/SLN/polymeric nanoparticles)	Enhanced follicular targeting for minoxidil/finasteride (preclinical/early clinical).	Promising to reduce dose frequency and systemic exposure; veterinary translation needed.	Controlled release; better follicular deposition.	Manufacturing/scale-up; regulatory and safety evaluation needed.
Microneedles/hydrogel-based controlled release	Microneedling-assisted delivery; hydrogel controlled release systems.	Could mitigate fur/SC barrier issues for localized sites; requires species-specific devices.	Improved penetration and residence time; lower systemic exposure potential.	Device cost; tolerability; need for veterinary protocol validation.
LAI and depot systems	Limited for alopecia in humans; concept used in other fields.	High potential to improve adherence in pets; active development trend in veterinary pharmaceutics.	Reduced dosing frequency; improves compliance.	Formulation and safety constraints; reversibility challenges if adverse events occur.

PRP, platelet-rich plasma; LAI, long-acting injectables; SLN, solid lipid nanoparticles; SC, stratum corneum.

## 5 Pharmacological and pharmaceutical comparisons of alopecia treatments in humans and companion animals

Building on the comparative framework established in the preceding sections, this section integrates pharmacological and pharmaceutical considerations that influence drug selection, pharmacokinetics, safety, and formulation strategies for the management of nonscarring alopecia in humans, dogs, and cats. Similarities in fundamental follicular signaling pathways provide opportunities for comparative research, but they do not justify direct therapeutic extrapolation across species. The dominant therapeutic target varies substantially according to both the underlying disorder and the affected species (56, 66).

In humans, pharmacological management frequently targets follicular miniaturization, androgen signaling, vascular support, or autoimmune inflammation (73, 135). Accordingly, minoxidil, 5α-reductase inhibitors, antiandrogens, corticosteroids, and Janus kinase inhibitors occupy central positions in the treatment of androgenetic alopecia and alopecia areata (73, 135). In dogs and cats, however, alopecia more often represents a clinical manifestation of an underlying endocrine, infectious, allergic, parasitic, hereditary, or behavioral disorder (13). Successful veterinary treatment therefore depends primarily on identifying and correcting the underlying cause rather than applying a nonspecific hair-growth stimulant (13).

### 5.1 Structural differences in skin and hair follicles

The human scalp has relatively thick epidermal and dermal layers, with hair follicles that are deeper and more sparsely distributed than those of most companion animals. In contrast, dogs and cats have thinner epidermal layers and higher follicular density. Their follicles are often grouped into clusters known as compound follicles in which one primary follicle is surrounded by multiple secondary follicles (50, 129, 136, 137). The diameter and length of hairs also vary; human terminal scalp hairs are longer and coarser, whereas canine and feline hairs tend to be shorter and finer (50, 138–141). These differences can affect not only drug absorption through the skin but also drug distribution within the follicular microenvironment (121). The barrier function of the stratum corneum also varies across species. Human skin generally has a higher lipid content and lower permeability than canine skin, which may increase the likelihood of systemic absorption of topical drugs in dogs (66, 130, 142).

### 5.2 Species differences in drug metabolism and ADME properties

Pharmacokinetic properties, including ADME, are critical determinants of drug efficacy and safety, and differ markedly between humans and animals. For example, cats are deficient in certain hepatic glucuronyl transferases which impair phase II metabolism, rendering them highly sensitive to drugs that rely on glucuronidation for detoxification, such as minoxidil (70, 71, 143). Dogs exhibit faster metabolic clearance of many drugs than humans, which may necessitate higher or more frequent dosing to maintain therapeutic levels (144–146). Furthermore, drug distribution is affected by differences in skin blood flow, follicular density, and body composition, all of which affect how drugs accumulate in the hair follicle niche (66, 121, 147).

The systemic bioavailability of orally administered drugs also varies. For instance, finasteride achieves predictable plasma levels and reduces scalp DHT in humans. However, although its pharmacokinetics in dogs have been characterized (101), its clinical efficacy remains unvalidated, partly because of species-specific differences in androgen metabolism and follicular androgen receptor expression. Similarly, the oral bioavailability of melatonin in dogs, and, to a lesser extent, cats, varies considerably between individuals (148).

### 5.3 Safety and adverse effect profiles

The safety profiles of alopecia treatments are highly species specific. In humans, finasteride and dutasteride are generally well tolerated, but can cause sexual side effects, such as low libido, erectile dysfunction, and reduced semen volume, due to the systemic inhibition of DHT (149, 150). In male dogs, however, administration of 5α-reductase inhibitors has raised concerns about reproductive toxicity, testicular atrophy, and reduced semen quality, limiting their potential veterinary application (151–153). Minoxidil is well tolerated in humans at recommended dose; however, in cats it can cause severe, even fatal, cardiovascular toxicity after dermal exposure, highlighting their extreme sensitivity to the drug (15, 154).

Topical vehicles and excipients may also contribute to these adverse reactions. Humans may experience contact dermatitis from propylene glycol-containing minoxidil solutions, whereas in animals, alcohol-based vehicles may exacerbate skin irritation and increase the risk of systemic absorption (15, 109). These observations emphasize the importance of formulating well tolerated, species-appropriate delivery systems.

### 5.4 Drug resistance and long-term use considerations

Long-term therapy for alopecia is often necessary to maintain clinical improvement, because treatment cessation usually results in relapse. In humans, chronic use of minoxidil or finasteride is generally considered safe. However, variability in long-term treatment responses has been noted, which may reflect ongoing follicular miniaturization or individual differences in androgen receptor signaling (155, 156). In veterinary medicine, little is known about the potential for long-term drug resistance or tachyphylaxis, partly due to the scarcity of controlled longitudinal studies. However, the development of dermal fibrosis or permanent follicular atrophy in certain forms of alopecia may render medical therapies ineffective over time.

Chronic suppression of androgen signaling raises concerns regarding hormonal imbalances and long-term safety; however, studies on companion animals are limited (153). In addition, owner compliance with long-term treatment regimens for pets can be problematic, particularly when therapies involve daily topical or oral administration (107).

In conclusion, although humans and companion animals share basic hair follicle biology, profound differences in skin structure, follicular morphology, drug metabolism, and pharmacokinetics dictate the need for distinct pharmacological and pharmaceutical approaches to alopecia treatment (51, 66). These differences affect not only efficacy but also safety, tolerability, and the feasibility of long-term therapy. Therefore, optimization of treatment in veterinary patients requires careful consideration of species-specific physiology, metabolism, and behavior, together with the development of formulations and delivery methods tailored to the needs of each species (66, 70).

## 6 Recent advances and innovative technologies in alopecia therapy

In recent years, growing insights into the molecular and cellular mechanisms underlying hair follicle biology have spurred the development of advanced and innovative therapies for alopecia. These approaches are designed to overcome the limitations of conventional pharmacological agents by targeting hair follicle stem cells, modulating the immune privilege of the follicle, and delivering drugs more effectively to the follicular niche. Emerging methods include regenerative medicine techniques, sophisticated drug delivery platforms, and personalized medicine strategies based on molecular biomarkers.

### 6.1 Stem cell therapy and regenerative medicine

Stem cell-based therapies represent a promising frontier in the treatment of alopecia. Hair follicles harbor a niche of multipotent stem cells within the bulge region that play a critical role in hair cycle regulation and regeneration (157, 158). Recent studies have explored the transplantation of autologous or allogeneic mesenchymal stem cells (MSCs) into the scalp, which can secrete paracrine factors such as VEGF, IGF-1, and Wnt proteins that activate resident follicular stem cells and promote angiogenesis (159, 160). Clinical trials have demonstrated improved hair density and thickness in patients with AGA treated using adipose-derived stem cell-conditioned media or MSCs (161, 162). Similar strategies are being investigated in veterinary dermatology; however, robust data on dogs and cats are currently lacking.

### 6.2 Platelet-rich plasma

PRP, an autologous concentration of platelets in the plasma, has gained popularity as a minimally invasive regenerative therapy. Platelets release growth factors such as platelet-derived growth factor (PDGF), transforming growth factor-beta (TGF-β), and VEGF, which can enhance follicular angiogenesis, prolong the anagen phase, and improve follicular stem cell activity (163). Multiple randomized controlled trials have shown that intradermal PRP injections lead to significant increases in hair count and thickness (163, 164). Although PRP has not yet been systematically studied in veterinary species for alopecia, it is increasingly being used to treat orthopedic and soft tissue indications in dogs and horses, suggesting its potential applicability in dermatology.

### 6.3 Low-level laser therapy

LLLT involves the use of red or near-infrared light at low power densities to stimulate cellular activity and enhance hair regrowth. Photobiomodulation at the follicular level is thought to improve mitochondrial activity, increase ATP production, and upregulate the Wnt/β-catenin signaling pathways critical for anagen induction (165, 166). Clinical studies have demonstrated the efficacy of LLLT devices in increasing hair density and reducing hair loss in humans. The use of LLLT in the treatment of dermatological conditions has also been described in dogs; however, controlled studies focusing specifically on alopecia are lacking.

### 6.4 Nanotechnology and smart drug delivery systems

Advances in nanotechnology have facilitated the development of novel drug delivery systems designed to enhance skin penetration and improve hair follicle targeting. Nanoparticles, liposomes, and solid lipid nanoparticles encapsulating minoxidil, finasteride, or natural compounds have demonstrated superior follicular delivery and controlled release in preclinical studies (167–170). Additionally, microneedle arrays, which create transient microchannels in the skin, have shown promise in facilitating the delivery of therapeutic agents and enhancing the efficacy of PRP and stem cell therapies (171, 172). These technologies also hold potential in veterinary application, where drug penetration through fur-covered and variable skin poses significant challenges.

### 6.5 Biomarker-based personalized medicine

The heterogeneity of alopecia, even within the same clinical subtype, highlights the need for personalized therapeutic approaches. Molecular profiling of hair follicles and serum markers has revealed potential biomarkers that are predictive of treatment responses, such as androgen receptor gene polymorphisms, DHT levels, and inflammatory cytokines (173, 174). These insights are paving the way for precision medicine strategies that tailor treatment to the individual patient's genetic, hormonal, and immune profiles. Biomarker discovery in companion animals is still in its infancy; however, ongoing genomic and transcriptomic studies in dogs and cats may eventually facilitate similar personalized approaches.

### 6.6 Veterinary clinical research and trends

While most cutting-edge therapies have been developed and tested primarily in humans, there is growing interest in adapting these treatments for use in veterinary medicine. Clinical trials in dogs have evaluated the efficacy of melatonin, nutritional supplements, and hormonal therapies in the treatment of various forms of alopecia (175, 176). PRP and stem cell therapies, already employed for orthopedic and wound-healing indications in dogs and horses, are being explored experimentally for dermatological use (177, 178). Pilot studies have described the use of LLLT for canine dermatosis; however, robust evidence is still lacking (179). The development of animal-specific devices, formulations, and protocols remains a crucial area of future research.

In summary, innovative therapies, such as stem cell transplantation, PRP, and LLLT, offer new avenues for treating alopecia by harnessing regenerative and immunomodulatory mechanisms. Advances in nanotechnology and smart delivery systems promise to improve drug targeting, efficacy, and patient adherence, whereas biomarker-guided personalized medicine could further optimize outcomes. Although veterinary applications of these technologies are still in their early stages, translational research and comparative studies hold great potential for extending these benefits to companion animals, ultimately improving human and veterinary dermatological care.

## 7 Future perspectives and clinical–pharmaceutical challenges in the development of alopecia treatments

Despite significant progress in the understanding and management of alopecia in humans, the development of safe and effective therapies for humans and companion animals remains challenging. Differences in the pathophysiology, pharmacokinetics, behavior, and clinical needs between species underscore the importance of tailored approaches that consider biological and practical constraints. Several key directions and challenges have emerged in translational, regulatory, and clinical research.

### 7.1 Commonalities and differences in drug development

Human and veterinary alopecia therapies seek to restore hair follicle function and promote regrowth, often targeting shared pathways, such as androgen metabolism, follicular stem cell activation, and angiogenesis. However, the biological significance of these pathways varies among species. In humans, androgen-driven follicular miniaturization is the central mechanism underlying AGA, the most common form of hair loss (73, 111). In contrast, alopecia in dogs and cats more frequently involves heterogeneous etiologies including hormonal dysregulation, immune-mediated processes, and idiopathic mechanisms (180–182). This divergence means that while therapeutic targets may overlap conceptually, specific agents and their mechanisms often require modification or alternative strategies in veterinary medicine.

Pharmacokinetic differences, such as the deficiency of glucuronidation pathways in cats or higher metabolic rates in dogs, also limit the direct translation of human drugs to veterinary settings (70, 143, 144, 183). Behavioral factors such as grooming and resistance to oral medications further complicate adherence and efficacy in animals. These differences highlight the need for dedicated preclinical and clinical testing in animal models rather than sole reliance on human data extrapolation.

### 7.2 Need for species-specific drug delivery systems and formulations

The development of species-appropriate drug delivery systems is an important frontier in veterinary dermatology. Topical therapies in humans benefit from open skin surfaces and patient cooperation, whereas the dense fur and self-grooming behavior of dogs and cats reduce the efficacy of similar approaches. Oral medications present compliance challenges, particularly in cats. Accordingly, interest in designing palatable chewable tablets, flavored suspensions, long-acting injectables, and pet-friendly topical formulations, is growing (16, 133, 184, 185). Advances in nanotechnology and sustained-release vehicles hold promise for veterinary use by reducing the frequency of administration and improving owner adherence (186, 187).

Furthermore, the development of smart drug delivery systems capable of targeting the follicular niche while minimizing systemic exposure could bridge the gap between efficacy and safety in humans and animals. Microneedles, liposomes, and biodegradable hydrogels are some of the technologies being explored in this context (170, 188–190).

### 7.3 Regulatory, safety, and ethical considerations

The regulatory landscape for alopecia treatment differs substantially between humans and animals. Human therapies must meet rigorous safety, efficacy, and manufacturing quality standards as mandated by agencies such as the FDA and EMA. Veterinary products face their own regulatory hurdles, often with more limited datasets and higher reliance on post-market surveillance (191). A key challenge in veterinary dermatology is the scarcity of controlled clinical trials, which hampers evidence-based approval and contributes to the widespread off-label use of human products in animals.

Safety concerns are of paramount importance, particularly in companion animals, where narrow therapeutic windows and unique metabolic profiles increase the risk of adverse effects. For example, the fatal cardiotoxicity associated with minoxidil in cats highlights the danger of extrapolating human doses to cats without species-specific testing (72). Ethical considerations also arise in veterinary research, including the need to minimize animal suffering, ensure owner consent, and justify the use of experimental interventions in line with the principles of the 3Rs (replacement, reduction, and refinement) (192, 193).

### 7.4 Multidisciplinary research and clinical trial expansion

Addressing these challenges requires a multidisciplinary approach that combines dermatology, pharmacology, veterinary medicine, materials science, and bioengineering. Collaborative networks among academic, clinical, and industrial partners can accelerate innovation and facilitate the translation of research findings across species. Expanded clinical trials in veterinary patients, designed to meet both regulatory and scientific standards, are essential for establishing evidence-based protocols and guiding appropriate dosing, formulation, and safety monitoring.

Advances in genomics, transcriptomics, and biomarker discovery further support the development of personalized medical approaches for alopecia, enabling clinicians to stratify patients based on their molecular profiles and predict treatment responses (194, 195). Continued investment in comparative dermatology and translational research will not only benefit veterinary patients, but also provide further insights into human hair disorders.

In conclusion, the future of alopecia therapy lies in the integration of biological understanding with pharmaceutical innovation, regulatory compliance, and ethical responsibility. Species-specific delivery systems, robust clinical evidence, and precision medicine approaches are central to meeting the diverse needs of both human and animal patients. Addressing these clinical and pharmaceutical challenges through interdisciplinary and translational research will improve the quality of care and therapeutic outcomes in the management of alopecia.

## 8 Conclusion

Alopecia in humans, dogs, and cats encompasses a heterogeneous group of disorders that share selected follicular mechanisms but differ substantially in their predominant etiologies, clinical manifestations, therapeutic targets, and levels of pharmacological evidence (13, 73). Human alopecia research has primarily focused on androgenetic alopecia and alopecia areata, leading to the development of treatments targeting androgen signaling, follicular growth, and immune-mediated pathways (73, 135). In contrast, alopecia in dogs and cats more frequently reflects underlying endocrine, hereditary, infectious, allergic, parasitic, or behavioral disease, and treatment must therefore prioritize etiological diagnosis and correction of the primary disorder (13).

Comparative analysis nevertheless offers important translational opportunities. Conserved signaling pathways involving Wnt/β-catenin, Sonic hedgehog, growth factors, immune privilege, and dermal papilla–stem-cell interactions provide potential targets for cross-species investigation (196, 197). Naturally occurring canine immune-mediated alopecia, hereditary follicular disorders, endocrine-associated hair-cycle arrest, and zoonotic dermatophytosis may also provide clinically relevant comparative models (13, 198). However, shared biological pathways do not establish therapeutic equivalence, and efficacy observed in humans cannot be assumed in dogs or cats without species-specific validation (66, 70).

Pharmacokinetic, metabolic, anatomical, and behavioral differences are particularly important for drug development (70, 199). Variations in follicular organization, coat density, skin permeability, hepatic metabolism, reproductive physiology, and grooming behavior influence drug absorption, retention, systemic exposure, safety, and adherence (66, 199). Veterinary formulations should therefore be designed specifically for the intended species and should minimize licking, oral ingestion, environmental contamination, and household cross-exposure.

Future research should emphasize well-controlled veterinary clinical trials, standardized outcome measures, breed- and season-specific evaluation periods, long-term safety assessment, and formulation strategies adapted to companion-animal use. A One Health framework that integrates human dermatology, veterinary dermatology, comparative pharmacology, and pharmaceutical science may improve both mechanistic understanding and the development of safer, more effective, and species-appropriate treatments for nonscarring alopecia (200, 201).
